# Improving the Performance of Phase-Change Perfluorocarbon Droplets for Medical Ultrasonography: Current Progress, Challenges, and Prospects

**DOI:** 10.1155/2014/579684

**Published:** 2014-06-01

**Authors:** Paul S. Sheeran, Paul A. Dayton

**Affiliations:** Joint Department of Biomedical Engineering, The University of North Carolina and North Carolina State University, Chapel Hill, NC 27599, USA

## Abstract

Over the past two decades, perfluorocarbon (PFC) droplets have been investigated for biomedical applications across a wide range of imaging modalities. More recently, interest has increased in “phase-change” PFC droplets (or “phase-change” contrast agents), which can convert from liquid to gas with an external energy input. In the field of ultrasound, phase-change droplets present an attractive alternative to traditional microbubble agents for many diagnostic and therapeutic applications. Despite the progress, phase-change PFC droplets remain far from clinical implementation due to a number of challenges. In this review, we survey our recent work to enhance the performance of phase-change agents for ultrasound through a variety of techniques in order to provide increased efficacy in therapeutic applications of ultrasound and enable previously unexplored applications in diagnostic and molecular imaging.

## 1. Introduction

Although the development of medical ultrasound imaging dates to the early 1900s [1], it remains a highly viable, widely used platform for anatomical/diagnostic imaging primarily because it affords noninvasive, nonionizing, and real-time information [2–4]. In the past 20 years, researchers have made further refinements in technology and manufacturing methods to advance the quantitative/diagnostic capabilities that a typical clinical machine can provide [5–8], and new therapeutic applications based on ultrasound are moving toward clinical implementation [9–11]. In parallel to these refinements in ultrasound technology, the field of ultrasound contrast agents (based on gas-core microbubbles) has continued to develop, enabling expanded possibilities for ultrasound-based diagnostics and therapeutics [10, 12–17].

Contrast enhanced ultrasound imaging currently differs from other modalities in that clinical microbubble formulations such as Definity [18] are inherently confined to the vasculature due to size. Although this intravascular limitation can be an advantage for agents that are specifically designed as blood pool tracers, it can also present a disadvantage when the desire would be to otherwise interrogate the interstitial space. Extravascular interrogation can be accomplished in PET/SPECT/MRI using micromolecular agents [19–21], where no such equivalent clinical extravascular microbubble agent exists for ultrasound. Clinical microbubbles also have fairly low circulation half-lives (on the order of minutes) due to gas solubility and natural clearance by the body (immune response, exhalation) [22]. To address these limitations, researchers have begun to increasingly investigate alternative contrast agents for ultrasound such as nanoscale bubbles, alternative encapsulations that increase bubble stability, echogenic liposomes, and phase-change perfluorocarbon (PFC) droplets [23–31].

Of these, phase-change droplets, or phase-change agents (PCAs), offer a unique solution in that the particles are designed to be metastable; the core is in the liquid state upon injection until activated by ultrasound energy to vaporize and expand, resulting in microscale gas bubbles. PCAs cores are typically composed of perfluorocarbons with boiling points near body temperature, such as dodecafluoropentane (DDFP, C_5_F_12_, boiling point 29°C) so that vaporization can be induced with low acoustic energies [32]. Though other compounds with similar boiling point, solubility, and molecular weight could be used, perfluorocarbons offer the benefit of a long history of use in medical imaging [33–35]. When designed properly, PCAs can exhibit greater stability in circulation and can be generated in a wide range of sizes [31], including nanoscale droplets that may be capable of interrogating the interstitial space of solid tumors via the enhanced permeability and retention effect [36, 37]. The drastic change in particle density, compressibility, and size when activated has resulted in a large number of applications in medical imaging and therapy that have high clinical potential. The historical background of PCAs, factors influencing vaporization, and an overview of the proposed applications are covered in several previously published review articles [31, 38–40] and are not repeated here. Despite the fact that phase-change agents have been under investigation for more than 15 years [41], and despite the high number of preclinical studies that have shown promise, no clinical studies (to the knowledge of the authors) have been conducted.

A thorough literature review of the current state of PCAs reveals several areas to fundamentally improve the clinical potential of the platform as follows.

- *Overcoming the increase in vaporization energy for nanoscale emulsions.* In some of the earliest publications surrounding PCAs, investigators assumed that droplets would vaporize once the ambient temperature was greater than the boiling point of the PFC core (typically DDFP) [42, 43]. Further investigation revealed that DDFP droplets not only remained in the liquid state once exposed to physiological temperatures, but only vaporized once exposed to temperatures at least 40°C above their nominal boiling point [44, 45]. In addition, experimental assessment of activation thresholds has revealed that droplets also required fairly significant acoustic pressures to vaporize that increase as droplet diameter decreases [32, 46, 47]. The delay in thermal vaporization may be in part due to the purity of the PFC allowing the droplet to exist in a superheated state for much longer than anticipated [48], but it is expected that Laplace pressure is one of the primary mechanisms of the increasing vaporization threshold as droplet size diminishes [31, 45]. The elevation in boiling point that results from this effect at the nanoscale is directly related to the acoustic energy required to vaporize a droplet. This explains the generally high vaporization thresholds reported for nanoscale PCAs of DDFP-activation pressures large enough in magnitude to prevent conventional formulations from being used for diagnostic and molecular imaging purposes [49–51]. Even for therapeutic purposes where higher ultrasound energy delivery into tissue is acceptable, this effect translates to a decrease in general efficiency of vaporization and may cause recondensation of particles after vaporization [52, 53]. Methods that produce PCAs more sensitive to ultrasound energy will be vital to increasing the effectiveness of PCAs in therapeutic applications and expanding their use toward diagnostic applications.
- *Increasing uniformity of activation.* Because the activation pressure of a droplet depends on the initial diameter, polydisperse distributions result in nonuniform activation. The techniques involved in preparing submicron emulsions typically yield polydisperse distributions, but for applications involving microscale droplets the uniformity of response (and therefore the effectiveness of the treatment) may be improved by creating monodisperse distributions through techniques such as microfluidic particle generation [54].
- *Tailoring droplet performance to desired application.* Nearly all the studies to date have investigated PCAs composed of a single perfluorocarbon [31, 39]. Although not well explored in the literature, the ability to manipulate the interplay of vaporization energy and thermal stability (resistance to spontaneous vaporization) for a droplet are essential in order to maximize the performance for a given application. As the range of applications widens, designing an agent that is optimally stable and one that vaporizes with ideal pressures may require a careful balance of competing factors. Choosing single perfluorocarbon species inherently limits the researcher to discrete points in the sensitivity/stability continuum, and so developing methods to more precisely “tune” droplets, such as PFC mixing [55], will enhance flexibility for clinical translation.
- *Determining appropriate activation thresholds for polydisperse submicron distributions.* Most studies aimed at determining appropriate activation thresholds* in vitro* and* in vivo* are either unable to resolve submicron particles (primarily optical methods) or are highly influenced by large outliers in the distributions (primarily acoustic methods). This makes assessing activation thresholds for the typically broad nanoscale distributions challenging and results in high variability in studies across different groups [39]. Alternative measures of activation thresholds or comparisons between measures are needed in order to determine* in vivo* activation conditions for polydisperse nanoscale emulsions.
- *Isolation of signals specific to PCAs for in vivo detection of activation.* The nonlinear oscillations produced by conventional microbubble contrast agents while under the influence of an ultrasonic pulse can be easily detected and isolated from tissue through a variety of techniques [56]. Developing similar detection techniques for PCAs to isolate the vaporization event from tissue and other ultrasound contrast agents will enable new forms of ultrasound contrast imaging and will allow spatial and temporal mapping of PCA activation to correlate with therapeutic and/or diagnostic goals.
- *Demonstration of utility for diagnostic and molecular imaging purposes.* Many studies have shown the utility of PCAs as therapeutic agents [31]. However, as a result of the relatively high vaporization thresholds for typical PCA formulations, few studies have shown that they can be used as purely diagnostic agents (i.e., their use as agents to generate imaging contrast) or molecular imaging agents. If vaporization thresholds can be reduced, demonstrations of the diagnostic potential of PCAs will greatly aid the push toward clinical use.

In the following paper, we review our recent work to develop solutions to the problems presented above and enhance the performance of phase-change agents for ultrasound. It should be noted that in some applications, it may be beneficial to produce emulsions that recondense and can be vaporized on subsequent pulses. Here, we frame our arguments in the more common assumption that stable bubble formation is ideal. We hypothesize that the techniques surveyed in this manuscript will provide increased efficacy in therapeutic applications of ultrasound, and may enable new approaches in diagnostic and molecular imaging.

## 2. Improving Uniformity of Response for Microscale Droplets

### 2.1. The Benefit of Microfluidics-Based Production

To date, PCAs have most commonly been composed of two perfluorocarbons—dodecafluoropentane and perfluorohexane (PFH, C_6_F_14_, boiling point 56°C) [31]. Researchers have used techniques such as sonication [45], extrusion [44], mechanical agitation [32], and microfluidization [47] to generate highly concentrated emulsions of these PFCs. These processes are capable of producing particles at both the nanoscale and microscale, although the emulsions are typically polydisperse. In order to ensure that large droplets in the distribution do not induce unwanted bioeffects, some studies have demonstrated further emulsion processing by filtering or microfluidic sorting [57–61]. Despite the refinement of the upper end of the droplet distribution, the polydispersity of the emulsion produces a decrease in the uniformity of response to the acoustic beam due to the size-dependent threshold of vaporization. Few options exist to reduce the polydispersity of submicron emulsions, but techniques such as differential centrifugation [62] or microfluidic generation [54, 63, 64] can be used to produce narrow distributions of microscale particles. Though microscale PCAs are not ideal as extravascular or imaging agents, they may have high utility in therapeutic applications where droplet vaporization produces bubbles large enough to occlude the blood vessels of target organs, increasing drug payload and residence time, inducing hypoxia, reducing the heat-sink effect of blood flow during thermal ablation, and acting as cavitation enhancement agents for ultrasound-mediated therapies [32, 65–67]. One of the earliest studies applying microfluidics technology to PCAs was by Couture et al. to create uniform loading of fluorescence in a composite PFC particle [54].

In three recent studies, we used the microfluidics platform to generate monodisperse microscale droplets of DDFP encapsulated in a phospholipid shell and demonstrate advantages and limitations of choosing this approach. In the first study, by Martz et al., a device previously designed for generating microbubbles with diameters near 10 *μ*m was adapted to produce monodisperse droplet distributions with diameters as low as 7.5 *μ*m in diameter at rates of 10^4^ to 10^5^ droplets/s [51]. A phospholipid solution was delivered via syringe pump to the outer channel (continuous phase) of the flow-focusing device, and DDFP was pumped into the inner channel (dispersed phase) via a separate syringe pump. By altering the flow rate of the two fluids relative, particles of different size were generated and collected. The benefit of monodispersity was demonstrated by testing vaporization thresholds of individual particles* in vitro* using a confocal acoustical/optical experimental setup. The different droplet populations (each representing a unique size with standard a deviation less than 5% of the mean diameter) vaporized at unique, nonoverlapping acoustic thresholds when subjected to 5 MHz ultrasound pulses (10 cycles)—confirming the hypothesized uniformity of response afforded by the narrow distributions. After collection, the particles stayed stable for more than 2 weeks in storage at 4°C. The authors concluded that the inability to generate monodisperse populations with mean diameters smaller than 7.5 *μ*m was a result of fluctuation introduced by the syringe pumps, and that this was especially prominent in the DDFP channel where viscosity was low and vapor pressure was high at room temperature.

**Figure 1:**
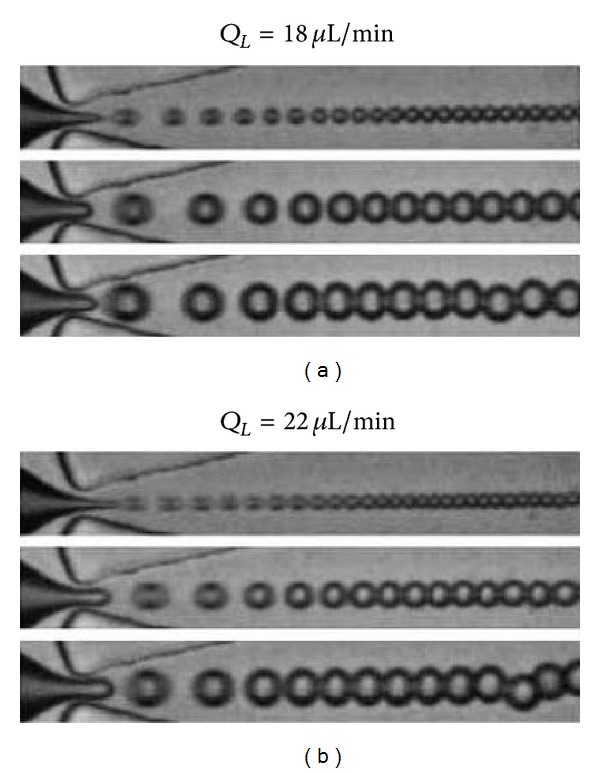
Sequence of images showing the effect of increasing (top-to-bottom) liquid dodecafluoropentane pump pressure for two phospholipid flows in the dripping regime. The droplet diameter decreases and the generation frequency quickens as *Q*_*L*_/*Q*_*P*_ increases. The image height is 25 *μ*m. Reproduced from [68] with permission from the Royal Society of Chemistry.

The second study, by Bardin et al., was performed in parallel and explored the relationship between reagent flow rates and droplet production regime in order to determine optimal droplet production parameters [68]. The authors demonstrated that by adjusting the flow rates, the microfluidic device transitioned between geometry-controlled droplet production (minimum droplet size determined by size of flow-focusing orifice), dripping production (capable of producing droplets smaller than the orifice), and jetting (producing droplets generally larger than the orifice). An experimental relationship between the droplet diameter and the reagent flow rates in the dripping regime of *D*_*d*_ = (*Q*_*c*_/*Q*_*d*_)^−5/12^ was developed, where *D*_*d*_ is the droplet diameter, *Q*_*c*_ is the continuous phase (phospholipid solution) flow rate, and *Q*_*d*_ is the dispersed phase (DDFP) flow rate. By switching the driving mechanism of the DDFP from a syringe pump to a more stable pressure-driven pumping mechanism, the authors were able to produce droplet samples with mean diameters on the order of 3 to 4 *μ*m before monodispersity was lost or a transition to the jetting regime occurred (Figure 1). Droplets were generated at similar rates to the above study by Martz et al. and stored at room temperature for two weeks with less than a 4% drift in size distribution—a promising result for future commercialization. Rather than vaporization with ultrasound, the authors tested the thermal vaporization point of the droplets. Though the nominal boiling point of the DDFP is 29°C, no vaporization was noted for 4.5 *μ*m diameter droplets until the ambient temperature reached 88°C. This high degree of superheat before vaporization may be mostly due to the high PFC purity, but may be in part due to a stabilizing influence of the encapsulation (via Laplace pressure). Once vaporized, the bubbles expanded to well over 100 *μ*m (presumably due to the previously reported intake of ambient dissolved gasses [30]) and settled to bubbles near 27 *μ*m in diameter once equilibrium was reached.

In the final study of this series, Martz et al. extended the use of pressure-driven flow to both the dispersed and continuous phase and increased the proportion of glycerol and Pluronic F-68 (Sigma Aldrich Corporation, St. Luois, MO) in the phospholipid solution [69]. Through this combination, the same device used in the previous studies was able to produce primary submicron droplets of an order of magnitude smaller than the minimum feature size of the microfluidic device. The authors hypothesize that these changes allowed the device to be operated in a “tip-streaming” regime [70], generating a fine spray of droplets as small as 300–400 nm in diameter. Although monodispersity was not preserved, the achievement of submicron droplet production without the use of techniques such as satellite droplet separation [71] may be useful for a wide range of medical and industrial applications. When exposed to 3.2 MHz ultrasound pulses (100 cycles), droplets near 0.9 *μ*m in diameter were vaporized at peak negative pressures near 4 MPa, while droplets much smaller than this were not able to be vaporized at the maximum output of the transducer (4.3 MPa peak negative pressure) as a result of the Laplace pressure effect.

### 2.2. Summary

These studies show that when the production method allows for a monodisperse size distributions, activation thresholds are highly uniform from droplet to droplet. For applications requiring microscale droplets (such as temporary vascular occlusion [57]), this will translate to an increased efficiency of vaporization* in vivo* compared to polydisperse distributions, increasing the effectiveness of the treatment and likely reducing the necessary dose. The results also show that the microfluidics-produced microdroplets were sufficiently stable to be stored at room temperature, which was unexpected given the nominal boiling point of the perfluorocarbon used. Despite this progress, a number of questions and future studies remain. For example, to produce the same number of particles as the alternative techniques (typically on the order of 10^9^ to 10^10^ particles per mL [46]), the microfluidic devices must be run for several hours at production rates of 10^4^ to 10^5^ droplets/s. Though this may be improved by scale-up and parallelization of the microfluidic device [72], it is unclear to what degree this is necessary; if the benefit of monodispersity is that droplet activation is highly uniform, then a fewer number of particles need to be injected to achieve the same effect as a polydisperse emulsion. Depending on this degree of improvement, it may be that the low production rates are sufficient, but future studies comparing monodisperse to polydisperse emulsions at similar concentrations or perfluorocarbon dose will be needed to determine this. Monodisperse microscale emulsions also present a unique opportunity to characterize some physical aspects present at the microscale: by creating distributions of different mean diameters and determining the phase transition temperature of these droplets; an experimental relationship between droplet size and vaporization temperature could be constructed and used to estimate Laplace pressure (and, therefore, surface tension) or the degree of superheat as a function of perfluorocarbon purity. Additionally, the microfluidics approach could be adapted to create PFC droplets with alternative encapsulations such as proteins or polymers. In summary, the microfluidics platform presents a powerful approach to optimizing current formulations of microscale PCAs for clinical implementation.

## 3. Tailoring Thermal Stability and Sensitivity to Ultrasound

### 3.1. Use of Volatile PFCs to Reduce Vaporization Thresholds

In order to circumvent the increase in vaporization thresholds that occurs as droplet size diminishes, we investigated methods of developing PCAs with highly volatile perfluorocarbon species that had not been previously considered in the literature. We first estimated the effect of Laplace pressure increase on size-dependent boiling point elevation for a range of perfluorocarbons through the Clausius-Clapeyron relation [50]. These calculations showed that two perfluorocarbons—decafluorobutane (DFB, C_4_F_10_, boiling point −1.7°C) and octafluoropropane (OFP, C_3_F_8_, boiling point −36.7°C) may experience a significant enough Laplace pressure at the nanoscale that the boiling point elevation is above physiologic temperature (37°C) depending on the droplet size and interfacial surface tension. Under these circumstances, liquid droplets of DFB and OFP might be stabilized at these sizes and remain in the liquid state once exposed to physiologic temperature until activated by ultrasound.

*In vitro* proof-of-principle was demonstrated by condensing DFB gas at reduced temperatures, placing an aliquot of the DFB liquid into a phospholipid solution and then extruding this combination through a porous membrane filter at −20°C to form phospholipid-encapsulated DFB droplets. The resulting emulsions were investigated with brightfield microscopy and were shown to contain DFB droplets as large as 13 *μ*m in diameter—much larger than predicted by the Laplace pressure estimations. Whether the presence of these large microscale droplets is a result of delayed vaporization (i.e., superheat of a pure compound) or a higher Laplace pressure than expected is unknown. Laplace pressure is not anticipated to be a significant influence at diameters on the order of 10 *μ*m unless surface tension is high, but the extremely elevated thermal vaporization thresholds shown in the microfluidics studies by Bardin et al. [68] suggest there may be some influence. Despite these questions, the results confirm that metastable droplets can be generated from compounds more volatile than previously considered. The vaporization thresholds of these microscale DFB droplets were tested using a microvessel-mimicking phantom at 37°C, and the results showed that when exposed to 5 MHz (10 cycles) pulses, DFB required significantly less pressure to vaporize than similar droplets of DDFP, and droplets of PFH were not able to be vaporized at the maximum output of the transducer. Droplets with diameters near 1 *μ*m (approaching the resolution limit of the setup) were able to be vaporized at peak negative pressures near 3.3 MPa—less than the maximum allowed for diagnostic imaging at 5 MHz (MI = ~1.5), while similarly sized droplets of DDFP exceeded this limit (on the order of 4.5 MPa; MI = ~2.0). Curve fits to the microscale experimental results were used to predict the vaporization threshold for submicron droplets of DFB, which remained below the limit for diagnostic imaging until droplets reached diameters smaller than 160 nm.

**Figure 2:**
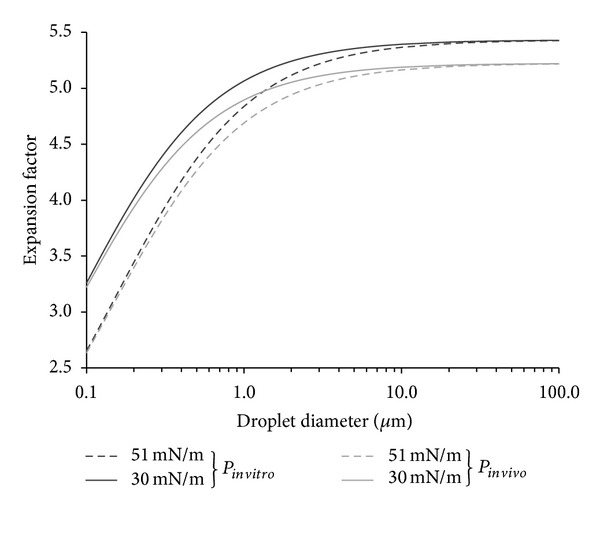
Effect of droplet size on expansion factor according to ideal gas laws with Laplace pressure included. Calculations are presented for two variations of both ambient pressure (*P*_*in* *vitro*_ = *P*_atm_; *P*_*in* *vivo*_ = *P*_atm_ + *P*_body_) and surface tension (*σ*_1_ = 30 mN/m; *σ*_2_ = 51 mN/m). Droplets on the order of 10 *μ*m can be expected to expand less* in vivo* than* in vitro* regardless of surface tension, while droplets of 500 nm or less will expand less at higher surface tension values. Reprinted from [50], Copyright 2011, with permission from Elsevier.

The experimental results with DFB and DDFP microdroplets also showed that, once vaporized, the level of dissolved gasses in the surrounding fluid plays a large role in the bubble size produced. PFCs are known to solubilize large amounts of oxygen, which has led to applications in liquid breathing and oxygen delivery [73–75]. This effect for PCAs was previously reported by Kripfgans et al. [30] and was confirmed here. Both DDFP and DFB bubbles expanded to nearly twice the size predicted by ideal gas laws (ideally 5-6 times the initial diameter) in the few seconds following vaporization. When the surrounding fluids were degassed, bubbles measured at the same time points followed ideal gas law predictions closely. This suggests that understanding the gas concentration of the intended* in vivo* target is important in understanding the behavior of the bubbles being generated by phase-change agents—especially for applications such as vascular occlusion. For PCAs to be used in diagnostic applications, emulsions that vaporize to form bubbles on the order of 1 *μ*m in diameter—similar to current clinical microbubble formulations—would be ideal. Theoretical expansion factors based on ideal gas laws including effects of surface tension and increased ambient pressure* in vivo* developed in this study show that droplet diameters between 200–300 nm should provide ideal bubbles once vaporized (Figure 2). However, droplets in this size range are larger than ideal for exiting the vascular space at solid tumor sides via the enhanced permeability and retention (EPR) effect (ideally diameters of 100 nm or less [36]), and so there may be a tradeoff between ideal bubble size for imaging and ideal droplet size for extravasation.

The extrusion method used to generate droplets did not appear to be capable of producing viable submicron droplets regardless of the membrane pore size used, presumably due to the very low surface tension of the PFC and the very high viscosity of the phospholipid solution at −20°C. Though future refinements to this approach may enable extrusion of submicron particles, we chose to explore alternative means of particle generation more amenable to the volatile compounds being investigated.

### 3.2. Microbubble Condensation: A Novel Approach to PCA Production

**Figure 3:**
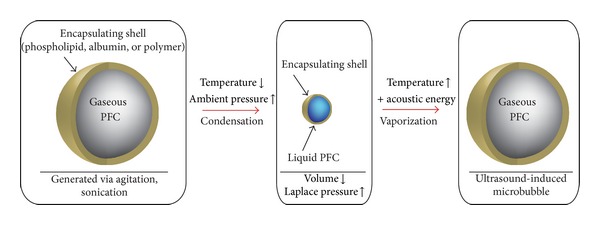
Exposing preformed PFC microbubbles to decreased ambient temperature and increased ambient pressure results in condensation of the gaseous core. The decreased size results in an increased Laplace pressure, which serves to preserve the particle in the liquid state. Once exposed to increased temperature and energy delivered via ultrasound, vaporization of the droplet core results in a larger, highly echogenic gas microbubble. Reprinted from [77], Copyright 2012, with permission from Elsevier.

Because both DFB and OFP exist as a gas at room temperature, producing liquid nanoscale particles through the conventional techniques (microfluidization, extrusion, and sonication) is not simple. However, current clinical formulations of microbubbles use mechanical agitation to generate phospholipid-encapsulated bubble distributions with mean diameters between 1 and 3 *μ*m [18]. In the next study of this series, we demonstrated that it was possible to generate submicron droplets with volatile PFC cores by first generating a population of perfluorocarbon microbubbles ideal for ultrasound interaction and then condensing the gaseous precursors to the liquid state through a combination of decreased ambient temperature and increased ambient pressure [76]. Once the particles condense to the liquid state, the reduction in size results in a submicron distribution of droplets, and the Laplace pressure may aid in stabilizing the droplets against reexpansion once exposed to physiologic temperature until the droplets are vaporized by ultrasound or heat. We refer to this simple, high-yield technique as “microbubble condensation” (Figure 3).

Approaching PCA production by this means has several advantages. First, because microbubble condensation begins from a population of microbubbles ideal for imaging, the bubbles produced after particle vaporization is of ideal size (assuming condensation and vaporization proceed optimally). Second, there are many published techniques to modify and functionalize microbubble shells to enable applications in molecular imaging and drug/gene delivery [78, 79]. Through microbubble condensation, these modifications can be applied to the precursor microbubbles, resulting in functionalized droplets. Third, the size of the droplets produced can be altered by changing the size distribution of the precursor microbubbles.

**Figure 4:**
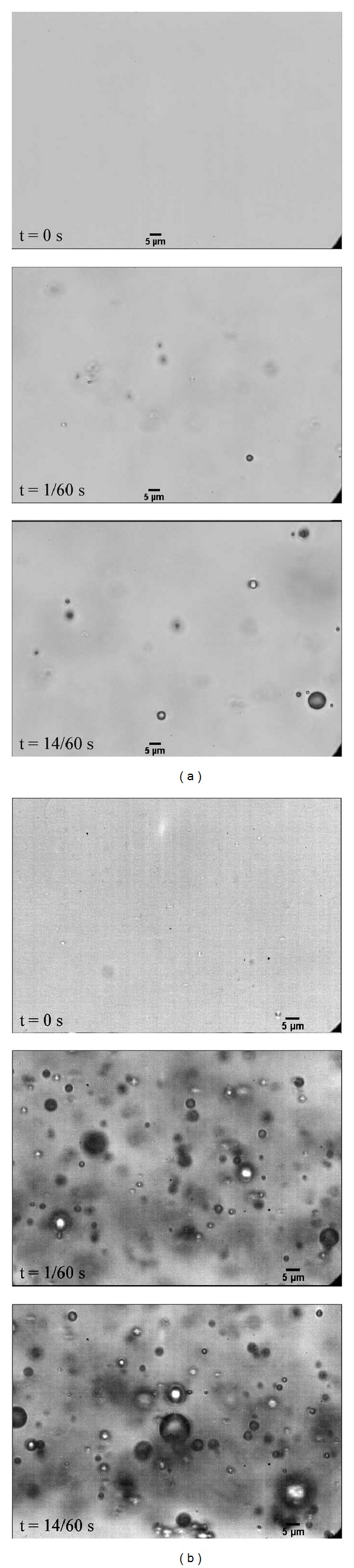
Droplet vaporization revealed microbubble sizes occurring predominantly in the 1–3 *μ*m range, with an increasing number of bubbles produced as lipid concentration increased. Vaporization was induced with a 10-cycle pulse at 5 MHz. (a) 0.75 mg/mL phospholipid concentration and (b) 3.0 mg/mL phospholipid concentration.* Reprinted with permission from *[76]. Copyright 2011 American Chemical Society.

In this study, we explored the changes in the precursor microbubbles and the condensation-produced droplets as a function of lipid concentration [76]. In all cases, microbubble condensation-produced droplets with distribution peaks on the order of 200–300 nm in diameter, as expected from ideal gas law predictions. When tested* in vitro* using the same experimental setup as in Section 3.1, few particles were visible in the imaging plane prior to application of the vaporization pulse, as droplets near 200 nm in diameter (below the wavelength of visible light) cannot be resolved in brightfield microscopy. Once a 5 MHz (10 cycles) pulse with a peak negative pressure of 3.8 MPa was delivered, the imaging plane was filled with bubbles on the order of 1–5 *μ*m in diameter (Figure 4). The amount of bubbles appeared to increase with the concentration of the lipid solution, but a larger amount of large microscale droplets was also observed at these concentrations—which may be unwanted (or cause unwanted bioeffects) in many applications. Finally, the bubble distributions at two different pressures (2.7 MPa and 3.8 MPa peak negative pressure) were compared to show that higher pressures produce a distribution with a higher proportion of small bubbles, indicating increased efficiency in vaporizing the emulsion and producing a distribution similar to the precursor microbubbles.

### 3.3. Fine-Tuning of Emulsion Properties by Perfluorocarbon Mixing

**Figure 5:**
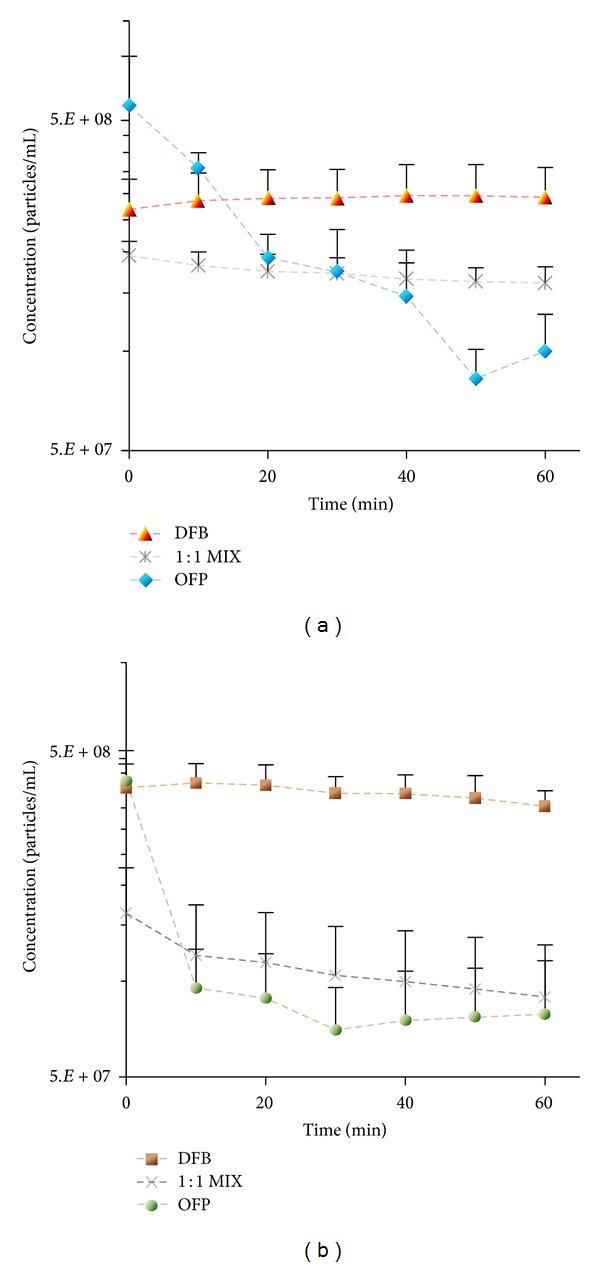
Change in concentration over time for droplet samples of each perfluorocarbon at (a) 22°C and (b) 37°C. Reprinted from [77], Copyright 2012, with permission from Elsevier.

In a subsequent investigation of microbubble condensation, we reveal the inherent tradeoffs involved with forming PCAs from volatile compounds [77]. Although it has been explored little in the literature, the gains made with regard to vaporization thresholds by increasing PFC volatility are likely to result in a decrease in thermal stability (as the vaporization pressure and temperature are governed by the Clausius-Clapeyron relation). This type of interplay has likely not been explored extensively in the literature due to the fact that droplets composed of higher boiling points (such as DDFP) are already sufficiently stable for most applications. The first portion of this study verified that microbubble condensation could be adapted to generate nanoscale droplets from highly volatile OFP. Some microscale droplets could be isolated* in vitro*, although it is expected that these exist in a highly superheated state and do not persist for long. By exposing both DFB and OFP microdroplets to ultrasound pulses at 8 MHz (2 cycles) at 37°C, vaporization thresholds could be compared. DFB droplets near 1 *μ*m in diameter were able to be vaporized at peak negative pressures near 2 MPa at 37°C and 3.5 MPa at 22°C, while similarly sized OFP droplets vaporized at peak negative pressures near 600 kPa at 37°C and 2 MPa at 22°C. Droplet stability was assessed by measuring the change in concentration and distribution of particles ≥500 nm in diameter at 10-minute intervals over the course of 1 hour (Accusizer 780A, Particle Sizing Systems, Santa Barbara, CA) (Figure 5). DFB droplets were generally stable* in vitro* during this period at 22°C and 37°C, while OFP concentration dropped significantly at both temperatures in just the first 10 minutes (an 80% decrease at 37°C).

**Figure 6:**
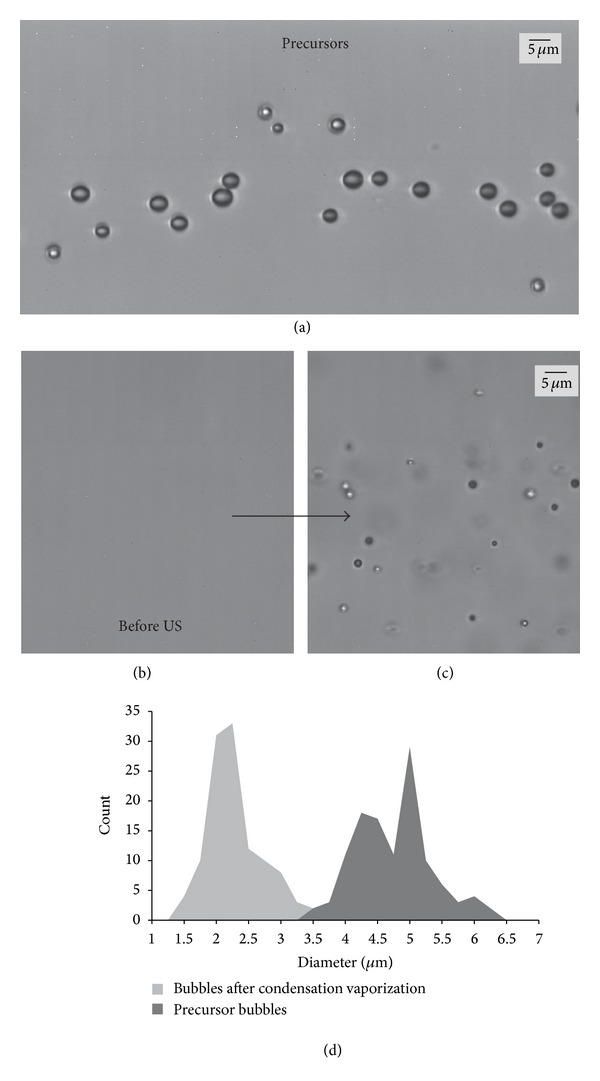
(a) Optical images of size-selected precursor microbubbles; (b) optical images of bubbles produced from vaporized droplets generated by microbubble condensation of the size-selected precursor bubbles; (c) histogram of shift in bubble distribution between precursor microbubbles (*N* = 116) and vaporization-produced bubbles (*N* = 118). Unpublished data.

These results demonstrate that, while choosing PFCs with lower boiling points produces droplets that are easier to vaporize by ultrasound, careful consideration must be given to the tradeoff in thermal stability that occurs in order to create agents capable of circulating for a sufficient period* in vivo*. Historically, researchers have designed droplets based on single perfluorocarbon species. Early PCA studies by Kawabata et al. showed that vaporization thresholds could be modulated by creating mixtures of the miscible perfluorocarbons [55]. We extend that to suggest that PFC mixing is a valuable tool to modulate both the thermal stability and the vaporization threshold in order to create droplets ideal for specific applications. Rather than choosing a “one PFC fits all” approach, the balance can be shifted in favor of higher stability or lower vaporization thresholds where appropriate. As a simple demonstration, we created droplets composed of a 1 : 1 mixture of DFB and OFP and showed that the resulting droplets had intermediate vaporization thresholds as well as intermediate stability (Figure 6). It is also important to note that PFC mixing is not limited to gas/gas or liquid/liquid, but that it is possible to mix across a wide range of PFCs that exist in different states in order to produce optimal droplet properties. In a separate study (detailed below in Section 5.2), we used a 1 : 1 mixture of DFB (gas at room temperature) and DDFP (liquid at room temperature) to create droplets ideal for ultrasound-mediated tissue ablation, where increased stability is desirable and higher vaporization pressures are acceptable [80].

### 3.4. Altering Droplet Distribution through Microbubble Size-Selection

One of the possibilities afforded by a microbubble condensation approach to PCA production is to alter the size of the droplets by changing the distribution of the precursor microbubbles. Methods of microbubble size-selection have been explored extensively and used to optimize particles for applications such as molecular imaging and blood-brain barrier disruption [62, 81, 82]. We have performed preliminary experiments showing that this approach can be adapted to produce altered PCA distributions (unpublished data). Adopting a protocol from Feshitan et al. [62] and Streeter et al. [82], size-selected DFB microbubbles were generated with an average diameter of 4.6 ± 0.6 *μ*m (*N* = 116, sized optically in the same degassed microvessel flow phantom from Section 3.1) at a concentration of 5 × 10^8^ bubbles/mL. After condensing the bubbles by the same protocol in Sheeran et al. [76], a dilution of 20 *μ*L droplet suspension in 1 mL of PBS was injected into the microcellulose tube and vaporized using an 8 MHz (2 cycle) pulse at peak negative pressures between 3.8 and 4 MPa. Before the vaporization pulse was applied, no droplets were apparent in imaging plane—implying successful condensation of the size-selected bubbles. After the vaporization pulse, a large number of bubbles of uniform size appeared on screen with an average diameter of 2.2 ± 0.6 *μ*m (*N* = 118). This change in average bubble size between the precursors and the bubbles after vaporization requires further discussion. In our prior studies, bubbles produced by droplets tended to be larger than the ideal gas law predictions due to gas influx in the bubble state [50]. However, when the environment was kept degassed, sizes were close to the expected values. In the microbubble condensation studies, the bubbles resulting from droplet vaporization were similar to the precursor distributions provided ultrasound pressure was high enough [76]. When vaporization pressure decreased, the distribution shifted toward larger bubbles. Here, the shift toward smaller bubbles from the precursors is unusual, and it is expected to be a result of a significant presence of air in the bubble in addition to the PFC. During size-selection, the microbubbles are exposed to the air interface for long periods of time, allowing some equilibration with the PFC content of the bubbles. During condensation, any room air that cannot be solubilized into the liquid PFC must be pushed out of the bubble in order for the liquid droplet state to be achieved—resulting in a decrease in bubble diameter once the PFC is fully vaporized again. Though sizing of the droplets was not performed, it is expected based on the vaporized bubbles that they existed in the 400–500 nm diameter range. It should be noted that the bubbles retained a narrow distribution despite the shift in mean size (see Figure 6). These preliminary results suggest that size-selection of PFC droplets may be possible with microbubble condensation, but that it requires limiting the presence of other gasses that may equilibrate with the PFC content of the precursor bubbles. It may also be possible to size-select the droplets once converted (i.e., differential centrifugation of the droplets rather than the precursor bubbles), though protocols to do this have not yet been defined.

### 3.5. Summary

In this section, we demonstrated new methods of fine-tuning the performance of PCAs with respect to ultrasound sensitivity, thermal stability, and size. Studies are ongoing to refine several aspects of these techniques, and many questions remain that will require future investigation. For example, in Sections 3.1 and 3.2, vaporization thresholds of microscale droplets were measured and used to predict the behavior of nanoscale droplets, although it is uncertain how accurate these predictions are. Recent studies have by Shpak et al. shown that frequency-dependent vaporization threshold effects exist for microdroplets due to microfocusing of the acoustic beam within the droplet [83], but these effects may not influence vaporization of nanoscale droplets at frequencies relevant to medical ultrasound, and therefore trends may not be predictive of nanoscale vaporization thresholds. Similarly, the concentration measurements used to determine thermal stability in Section 3.2 were measures of the larger content of the droplet distribution (particles > 500 nm in diameter). Whether the stability characteristics of this portion of the distribution accurately represent the stability of smaller droplets present is uncertain. For example, the OFP droplets 500 nm or greater in diameter were unstable at 37°C (Figure 5), but it may be that OFP droplets with diameters near 100–200 nm may be sufficiently stable. Also, it is simple to assume that spontaneous vaporization is the primary mechanism of degradation in the droplet emulsion, but it may be that a significant amount of dissolution occurs at the PFC/water interface for volatile perfluorocarbons—resulting in a gradual decrease in particle size until completely dissolved. It is possible that spontaneous vaporization is more common at the microscale where Laplace pressure is low, but that particle dissolution is more prominent at the nanoscale where internal pressure is high and the surface area-to-volume ratio has increased.

Other physical aspects of PCA production require further investigation. For example, the actual surface tension for different PCAs encapsulations—which determines the degree of Laplace pressure—is generally unknown and is largely dependent on the state of the encapsulating shell (i.e., likely to very different pre- versus post-vaporization). Determining this parameter for different shell types will be important in optimally stabilizing droplets made from volatile PFCs. In most approaches to phase-change agent generation, the particles are encapsulated optimally in the droplet state and vaporized to form gas bubbles. Some studies, including our extrusion-based results, have shown that the shell appears to be present after vaporization—allowing for stabilized bubbles that interact with ultrasound in a similar fashion to typical clinical microbubble formulations [50, 84]. The state of the shell at these much larger sizes is currently unknown but is likely to be sparse. Similarly, the change in conformity of the shell during microbubble condensation is currently unknown. In this method, particles are optimally encapsulated in the gas state and then converted to the liquid state. It may be that the phospholipid shell buckles in the PFC liquid state and reincorporates upon vaporization, but it is more likely that some of the lipid shell is shed during the process. Future studies are needed to determine whether this is the case and to determine what modifications must be made to maximize shell retention and optimize performance* in vivo*. Such changes in the encapsulating shell may have a significant impact on the ability to develop functionalized droplets (for drug/gene delivery or molecular imaging). It is possible that neither extreme (droplet encapsulation versus bubble encapsulation) is optimal, but that the shell must be designed to exist somewhere between both states. For example, recent studies by Sirsi et al. have examined the use of microbubbles encapsulated in lung surfactant [85] designed to fold during bubble compression and reincorporate upon bubble expansion. Alternatively, encapsulation materials such as albumin that thicken at the droplet state and thin to form ideal bubble shells upon vaporization may be preferable.

Future work will focus on refining the condensation protocol itself and verifying aspects of the approach. Estimates of concentrations for nanoscale emulsions are usually calculated by combining knowledge of the perfluorocarbon volume used with a number-weighted distribution [86]. In a condensation-based approach, this is not straightforward, as some of the perfluorocarbon volume persists in the gas headspace of the vial. Assuming a perfect 1-to-1 conversion of bubble to droplet would produce droplet concentrations on the order of 10^10^ particles/mL, which may be appropriate for perfluorocarbons such as DFB that require very little pressure to condense. The validity of this assumption becomes weaker for droplets composed of highly volatile compounds such as OFP, where higher ambient pressures are required to achieve condensation. However, relatively new technologies such as the qNano (iZON Ltd., Cambridge, MA) and the NanoSight 500 (NanoSight Ltd., Amesbury, UK) may circumvent the need to make such assumptions by offering a means to measure both the concentration and size distribution of nanoscale PCAs [87]. Finally, it may be possible that some portion of the remaining perfluorocarbon headspace condenses on exposure to reduced temperature and increased pressure, causing unwanted droplet formation. If so, this can be controlled by replacing the headspace with a lower boiling point gas that will not solubilize into PFC and then proceeding with condensation.

Regardless of the remaining questions, microbubble condensation appears to be a useful approach to producing submicron perfluorocarbon droplets. A recent study by Seo and Matsuura has cleverly combined a modified version of this technique with microfluidics to generate submicron DDFP droplets [88], and other research groups are beginning to investigate DFB droplets for unique applications in gene transfection and monitoring of vaporization [89, 90].

## 4. Improvements in Methods of Measurement, Detection, and Activation

### 4.1. Measuring the Activation of Polydisperse Nanoscale Emulsions

Developing an all-inclusive droplet vaporization model to accurately predict vaporization thresholds remains a challenge due to the wide range of factors (many summarized in [31]) that influence the likelihood of droplet vaporization. As such, methods to measure vaporization thresholds typically take two forms—physical (e.g., optical verification of individual droplet vaporization events at the transducer focus) or phenomenological (e.g., measure of ultrasound backscatter produced after a vaporization pulse has been delivered). As a result of the differences in these measurement techniques and the change in experimental choices from study to study, a wide range of reported thresholds exist with no “gold standard” of measurement to compare to. For nanoscale emulsions, both the physical and phenomenological approaches are limited for the typically broad distributions. In physical (optical) methods, the initial size of the droplets is below the wavelength of visible light and therefore unresolvable. Phenomenological methods are highly skewed by the presence of large outliers in the distribution, and so associating the measurement with a representative size is not straightforward. Thus, there is a need for improved measurements of activation for nanoscale emulsions.

**Figure 7:**
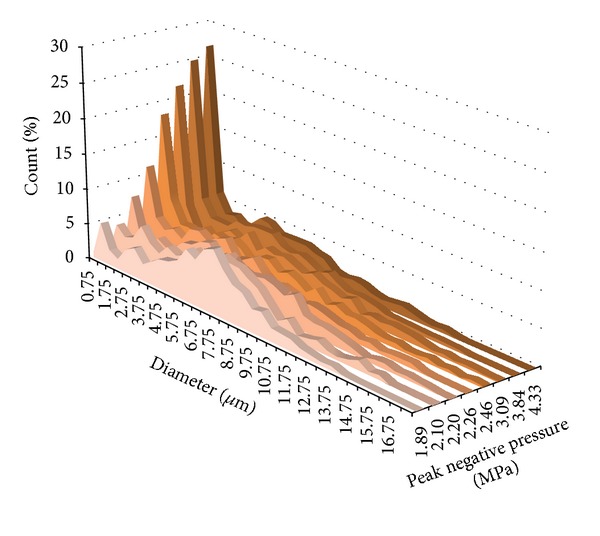
Histogram of all bubble sizings taken at 5.5 MHz (*N* = 11,654) as a function of rarefactional pressure. As pressure increases, the number of small bubbles increases in proportion until the smallest bin size overtakes as the peak in the distribution. Continued increase in the pressures amplifies the proportion of these small bubbles relative to other bubbles present in the distribution. Reproduced from [91] with permission from the Institute of Physics in Engineering and Medicine.

In a recent study [91], we expanded on previous results [76] to show that the shift in the bubble distribution produced as peak negative pressures increased could be used as an indicator of optimized vaporization. By relating the droplet and bubble size with ideal gas laws (as in [50]), droplets near 200–300 nm in diameter should vaporize to produce bubbles near 1 *μ*m in diameter. At low peak negative pressures, only the largest droplets in the emulsion vaporize, producing a bubble distribution with a mode much higher than 1 *μ*m. Once peak negative pressures are high enough to vaporize a significant amount of the 200 nm droplets, the mode of the bubble diameter distribution should reflect this by shifting to a value near 1 *μ*m. We demonstrated this* in vitro* at 1, 5.5, and 8 MHz (2 cycles) and showed that the transition point of 1 *μ*m bubble production could be captured experimentally (Figure 7). Interestingly, the transition peak negative pressure appeared to increase with ultrasound frequency. This result possibly reflects the influence of narrowing beam width at higher frequencies—requiring higher pressures to produce similar bubble distributions over the same spatial span and at the same droplet concentration.

Although the bubble distribution tracking method is capable of capturing the activation of nanoemulsions, the measurements are fairly labor-intensive. The study above measured the diameter of approximately 30,000 bubbles across all frequencies and pressures in order to determine the distribution shifts. In the future, these measurements could be automated to some degree, but it may be possible to combine a small set of distribution tracking tests with a simpler measurement method to reduce the work involved. For example, simultaneously capturing the change in bubble distribution and the echo intensity for a subset of the total tests would allow correlation between the transition point in the bubble distribution shift and the echo intensity that corresponds to this. The remaining tests could be carried out with the simpler measure of echo intensity once this transition point is calibrated. Another alternative was recently presented by Xu et al. that captured the decay in acoustic intensity produced from vaporization of unencapsulated DDFP droplets and related the decay to estimates of the underlying bubble distribution [92]. If this can be adapted to encapsulated droplets, it presents an interesting acoustic method to assess similar shifts in the bubble distribution as our microscopy-based approach.

### 4.2. Capturing Acoustic Signatures of Droplet Vaporization

Another possibility for measurement of droplet activation comes from a study showing that under certain circumstances the vaporization event can produce unique size-dependent acoustic behavior [93]. Although a wealth of techniques exists to differentiate microbubble signals from surrounding tissue based on nonlinear behavior, very few exist to differentiate PCAs from both tissue and microbubble signals. While in the liquid state, PCAs are only weakly echogenic and difficult to distinguish over tissue, but once vaporized to form bubbles, they produce signals similar to conventional microbubbles [84]. Some have suggested that the pressure wavefront emitted from a vaporization droplet could be distinguished acoustically [94], while others have proposed that the change in acoustic signals as the vaporizing bubble evolves could distinguish PCA-produced bubbles from those already present [95].

**Figure 8:**
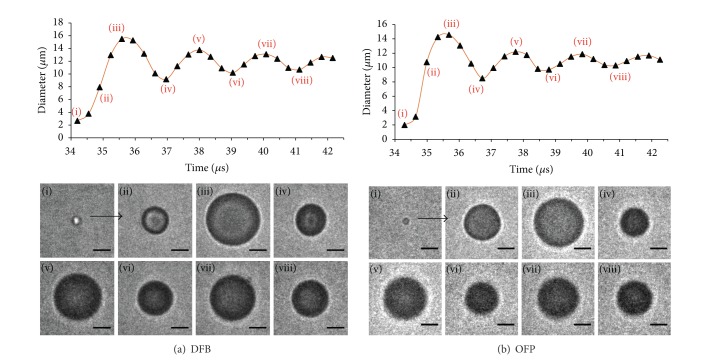
Vaporization and expansion properties of volatile PFC droplets vaporized with a 2-cycle sinusoid at 8 MHz. (a) A DFB droplet near 2.7 *μ*m in diameter vaporizes and expands to a maximum near 15.5 *μ*m in diameter within 2 *μ*s and eventually settles to a smaller resting diameter. (b) An OFP droplet near 2 *μ*m in diameter expands to a maximum near 14.6 *μ*m in diameter and settles to a smaller resting diameter. In both cases, the droplet oscillations occurred over the course of 10 *μ*s after vaporization. Scale bar represents 5 *μ*m. Reproduced from [93] with permission from the Institute of Physics in Engineering and Medicine.

In preliminary ultrahigh-speed imaging of droplet-to-bubble expansion, we noticed that when activated, DFB droplets oscillated well after the end of the activation pulse. Further investigation showed when short pulses (8 MHz, 2 cycles) are used to initiate bubble nucleation (but end shortly thereafter), the particle overexpands past the final bubble size and returns to the resting size in an oscillating manner (Figure 8).

This overexpansion/oscillation behavior was observed for droplets of both OFP and DFB, and measurements of the video data over many droplets showed that the relationship between particle size and oscillation frequency matched the expected relationship for unforced resonance of a bubble (modelled by Minnaert resonance [96])—with higher frequencies being produced by smaller particles. In this case, the droplet volatility appears to be sufficient to temporarily drive the growth of the bubble past its final diameter, and the overexpansion initiates the oscillatory behavior as the bubble returns to a final resting size. In the study, a damped harmonic oscillator model was applied in order to estimate the total damping coefficient. The expansion velocity, maximum overexpansion, and final bubble size were shown to increase with PFC volatility. The larger final bubble size for OFP particles compared to DFB particles was expected based on ideal gas laws, but the higher expansion velocity and maximum overexpansion observed for OFP is expected to be a result of the much higher degree of superheat.

**Figure 9:**
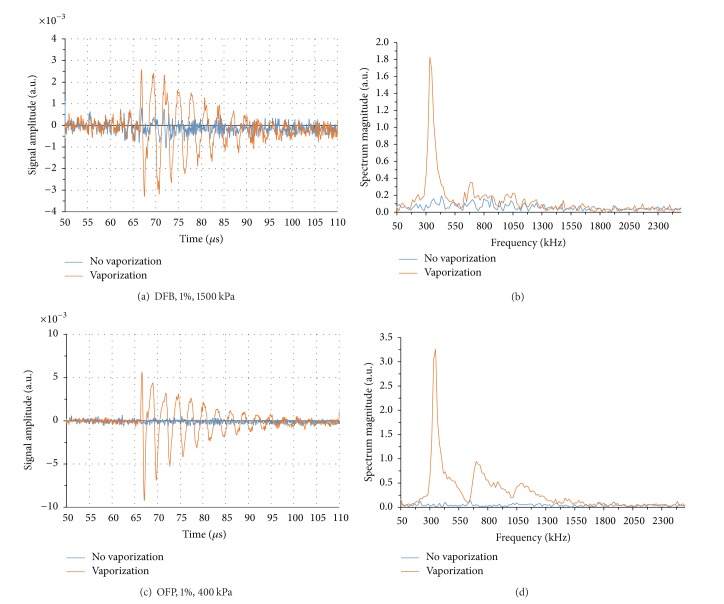
Signals produced by individual droplets vaporizing in the received transducer's focus manifest as exponentially decaying sinusoids with very narrowband frequency content. Examples of (a) DFB droplet vaporization, (b) the associated frequency spectrum, and (c) and (d) OFP droplets oscillating at similar frequency produce larger amplitude oscillations even when exposed to significantly lower incident pressure as a result of greater droplet volatility. In all graphs, the blue trace shows the preceding pulse that did not produce a droplet vaporization event for the same sample and pulse pressure. Reproduced from [93] with permission from the Institute of Physics in Engineering and Medicine.

The physical oscillations of vaporized particles displace nearby fluid, producing new acoustic waves (not related directly to the vaporization pulse) that can be detected by an ultrasound transducer. In the study, the acoustic emissions of vaporizing droplets were detected passively with a 1 MHz transducer (vaporization was initiated with an 8 MHz, 2-cycle pulse) and differentiated from signals produced under similar circumstances by microbubble contrast agents. As expected from the physical characterization, droplet vaporization manifested as exponentially decaying sinusoids in the time domain and as narrowband peaks in the frequency domain (Figure 9). Signals produced from OFP droplets were larger in magnitude than those produced by DFB droplets as a result of greater expansion and oscillation velocities. Both the frequency of oscillation and the signal amplitude were shown to be dependent on the initial droplet size, suggesting acoustic detection of this vaporization behavior may be a means of discerning whether droplets of specific sizes are being activated. We followed this with demonstration of a simple droplet-detection algorithm that measures phase-transition of droplets greater than approximately 1.4 *μ*m in diameter as a function of peak negative pressure and droplet concentration. This approach could conceivably be altered to measure activation of much smaller droplets provided the activation pulse does not overlap with the oscillation period.

Many aspects of this method of droplet detection are currently being explored. The rate of exponential decay of the droplet oscillations is directly related to the influence of the encapsulating shell, and much can be learned of the underlying physics from these measurements. There is currently only one vaporization model published in the literature that predicts this type of oscillatory behavior [97], and so comparing this with the experimental results or developing new models will be important in understanding specific aspects of the oscillation. For example, no other studies to date based on DDFP droplets, including the study by Qamar et al. [97], have experimentally observed these oscillations, which suggests there is a minimum degree of superheat needed to cause the overexpansion behavior. Additionally, Qamar et al. show that the oscillation behavior may not start for a given PFC until droplets are below a threshold diameter. With a greater understanding of droplet expansion physics, more robust detection algorithms may be implemented than those proposed in Sheeran et al. [93].

Perhaps the most interesting extension of the study is to implement the droplet signal detection on linear arrays and create droplet-specific imaging techniques capable of localizing activation both temporally and spatially* in vivo*. The PCA signals occur at relatively low frequencies and only travel one-way through the tissue to reach the transducer, and so it is likely that the signals can be captured* in vivo* provided droplets can be activated at the desired depth. There may be a practical limit to the minimum droplet size able to be detected, as the vaporization pulse must remain short enough to not drive the bubble oscillation. For droplets near 200 nm in diameter, this means that frequencies higher than 8 MHz must be used, which will provide limited depth of penetration. Another possibility is to use other modalities to vaporize the droplets (such as photoacoustics) that inherently do not drive the oscillation and will likely allow very high SNR capture of the emitted signals.

### 4.3. Developing Activation Pulses to Maximize Bubble Production and Minimize Bioeffects

Our study on measuring shifts in bubble distributions [91] also demonstrated preliminary evidence that the ongoing cycles of the vaporization pulse (after the droplet has begun vaporizing) can highly influence the resulting bubble distribution. Droplets vaporize during the rarefactional phase of the ultrasound but can be strongly affected by the subsequent compression and rarefaction phases. Small bubbles (such as those generated from nanoscale droplets) tend to be destroyed by the subsequent compression phases of the vaporization pulse, while large bubbles (such as those produced by microscale droplets) tend to translate toward each other due to secondary radiation force over several cycles and can fuse during rarefactional cycles. The likelihood of these types of effects increases with the pulse length, and so long pulses will likely result in a change toward larger bubble distributions. For diagnostic imaging this is generally undesired and pulse lengths must be as short as possible to limit the effect, but it may be a useful property for many therapeutic applications. In either case, a more complete understanding of the thresholds and impact of this behavior is needed in order to optimize the vaporization waveforms for the intended application.

### 4.4. Summary

In this section, we have demonstrated new techniques to measure droplet activation thresholds and capture new acoustic signals produced by droplets and proposed methods to optimize vaporization waveforms. We envision that the combination of these will allow more accurate assessment of the* in vivo* pressures required to activate nanoscale emulsions, greater efficiency of activation, and new PCA-specific imaging techniques.

## 5. *In Vitro* and* In Vivo* Demonstrations

Sections 2, 3, and 4 explored methods of improving PCA performance through a variety of* in vitro* techniques. In this section, we summarize our* in vitro* and* in vivo* work highlighting the new possibilities in therapeutic and diagnostic ultrasound afforded by these improvements.

### 5.1. *In Vitro* Demonstration of PCA Molecular Imaging

**Figure 10:**
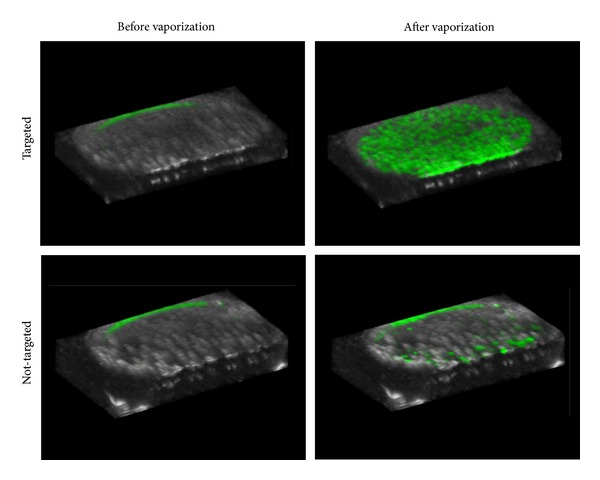
Overlays of contrast-specific CPS intensity (green scale) and traditional B-mode (grey scale) ultrasound scans: 3-D rendering of the ultrasound slices across the cell sample volume reveals the contrast enhancement provided by targeted PCAs after the activation pulse. Non-targeted samples resulted in a small amount of contrast enhancement, likely as a result of non-selective binding. The small amount of contrast present at the top of each sample is an artifact of the metal holder, not a result of the presence of echogenic bubbles. Reprinted from [99], Copyright 2013, with permission from Elsevier.

The reduction in vaporization thresholds through use of volatile compounds opens the possibility of using PCAs as purely diagnostic contrast agents. Current ultrasound approaches to molecular imaging modify the microbubble shell to include targeting ligands that attach to vascular molecular expression (such as angiogenesis). The attached bubbles are then differentiated from the freely circulating bubbles to gauge the level of molecular expression [79]. Previous studies have explored the use of targeted DDFP droplets, but it was found that the high vaporization pressures required to activate the droplets tended to destroy the cells, restricting use to therapeutic/theranostic purposes [98]. Targeted droplets that are able to be vaporized at diagnostic pressures may have high utility for ultrasound molecular imaging purposes—both intravascular and extravascular. We performed a simple* in vitro* proof-of-principle that DFB droplets modified to contain cyclic RGD peptides in the encapsulating shell can preferentially attach to expression of *α*_v_ *β*_3_ in the HUVEC cell line compared to sham controls containing cyclic RAD peptides [99]. As shown in [77], droplets produced by microbubble condensation retain the lipid encapsulation, and so targeted droplets can be generated by condensation of targeted precursor bubbles. The targeted and nontargeted droplets were incubated for 15 minutes with HUVEC cells, the cell samples washed and then activated by scanning a diagnostic ultrasound transducer (Siemens 15L8 linear array transducer) over the cell layer at 8 MHz with a nominal mechanical index of 1.1 (approximate peak negative pressure of 3.1 MPa). Prior to the vaporization sequence, very little bubble signal was present in the cell layer (measured using a contrast-specific imaging mode) for both droplet types, indicating that any droplets present remained in the weakly echogenic liquid state. After vaporization, a high level of contrast was observed in the targeted droplet samples along the cell monolayer (6-fold increase over nontargeted and 54-fold increase over baseline) (Figure 10).

Interestingly, the bubble appeared to remain adherent to the cell monolayer rather than detaching and drifting to the top coverslip, suggesting the shell remained intact enough to preserve targeting. Although the results provide preliminary evidence that molecular imaging with droplets is possible, whether this will be the case under flow conditions, and how this can be mitigated by altering shell composition will be the subject of future* in vitro* and* in vivo* tests. The droplet-based approach may be advantageous compared to microbubble-based molecular imaging in that the degree of molecular expression is immediately apparent after the vaporization pulse; any nontargeted or nonassociated bubbles will leave the imaging plane quickly and unvaporized droplets in the plane will not produce significant contrast. Optimizing this technique* in vivo* may require development of new droplet processing protocols to ensure that targeted lipid vesicles do not remain in the droplet suspension after condensation (which will block viable target sites) and will likely require development of custom droplet-based activation/imaging sequences on diagnostic ultrasound machines.

### 5.2. *In Vitro* and* In Vivo* Demonstration of HIFU Ablation

Perfluorocarbon phase-change agents have been proposed to increase the efficiency of high-intensity focused ultrasound (HIFU) procedures in numerous publications [31]. The primary advantage of their use is that droplet vaporization occurs in the region of highest ultrasound energy, and then the newly produced bubbles increase the cavitation activity and interaction with the tissue. In three studies, we showed that the concept of PFC mixing is a useful tool to refine the performance for HIFU therapy* in vitro* and* in vivo*.

**Figure 11:**
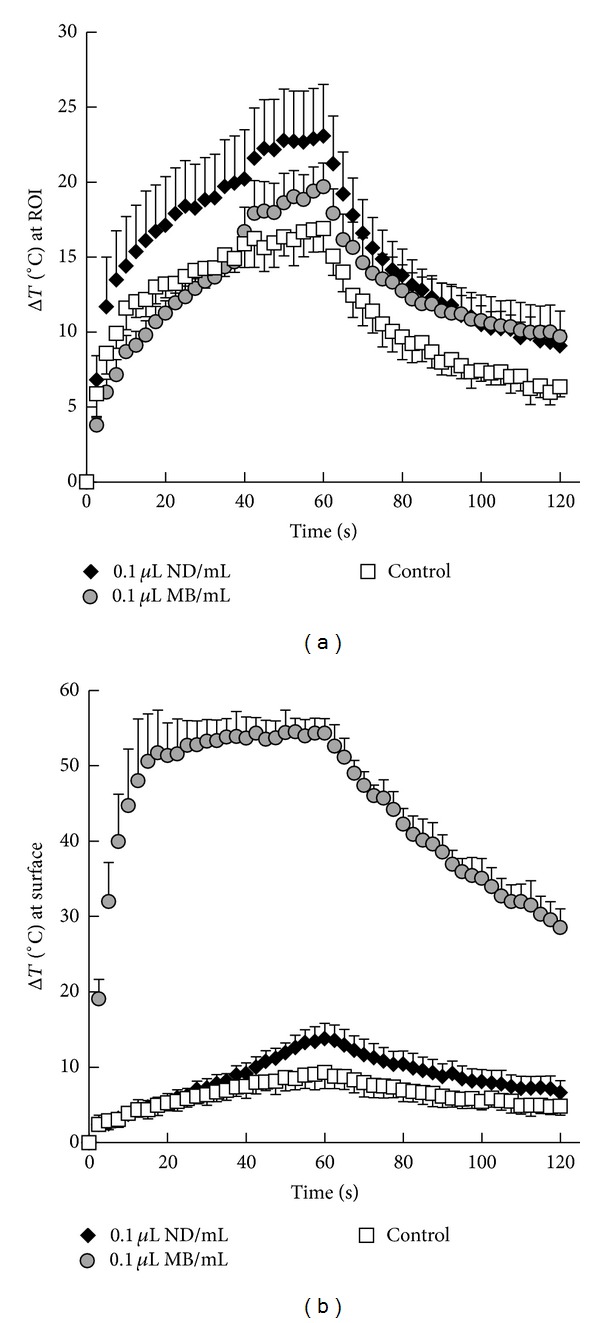
For each MR acquisition over time, the maximum change in temperature (a) at the focus (ROI) and (b) surface of the phantom was extracted from the corresponding horizontal slices from those locations. Time curves were averaged, and data are presented as the means ± s.d. (*n* ≥ 3). Phantoms contained 0.01 *μ*L of nanodroplets or microbubbles per milliliter of phantom material or no agents (control). Reprinted with permission from Phillips et al. [80]. Copyright 2013, Acoustic Society of America.

The first of these studies, by Phillips et al., investigated microbubble-condensation-produced droplets composed of a 1 : 1 mixture of DFB and DDFP [80] in tissue-mimicking phantoms. In parallel studies, it was determined that DFB droplets activate efficiently at pressures near 2 MPa using frequencies near 1 MHz [91]. Although DFB is generally stable over the course of 1 hour* in vitro* at physiologic temperatures, HIFU procedures can often last for several hours [100, 101]. Therefore, we sought to design a droplet with vaporization thresholds near 4 MPa that had high* in vitro* stability. By similar tests as in those used in [77], a 1 : 1 mixture of DFB and DDFP was shown to produce droplets stable for more than 48 hours when exposed to 37°C* in vitro*. Lesion formation with these droplets was studied using an acrylamide-albumin phantom that visibly denatures once temperature rise is similar to that used* in vivo* for tissue ablation. Tests with this phantom showed that the composite droplets enhanced lesion formation over agent-free controls at the desired ultrasound pressure levels (1 MHz, 4 MPa peak negative pressure). Compared to similar concentrations of microbubbles, which interacted strongly with the acoustic beam prefocally and led to unwanted lesion formation and surface heating at even low concentrations, droplets were better able to preserve the focal nature of the lesion formation. This was confirmed in MR thermometry experiments showing that microbubbles caused a high amount of heating at the surface of the phantom, while droplets generally maintained heating at the desired focus until concentrations were very high (Figure 11). The authors concluded that “…ablation lesions produced in the presence of nanoscale droplets were less prone to shape change and lesion migration than microbubble-enhanced lesions.” This highlights what is potentially one of the biggest advantages of PCAs in therapy; compared to microbubbles, PCAs may preserve focal aspects of therapy better, maximizing the effect where intended and minimizing it elsewhere.

The second study in this series, by Puett et al., explored the transition point in droplet concentration and acoustic parameters where control over the lesion formation was lost using the same* in vitro* tissue mimicking phantom [102]. Droplet concentrations ranging between 10^5^ and 10^8^ droplets/mL of phantom were tested, with pulse lengths ranging from 5 to 20 seconds of continuous-wave ultrasound at intensities of 140, 390, and 650 W/cm^2^. The study found that given a concentration of droplets distributed throughout the tissue, an appropriate pulse length and intensity could be chosen in order to maximize lesion size and minimize treatment time while maintaining high control over the lesion formation (determined by change in lesion shape and centroid). The study also demonstrated that vaporization of the droplets produces a bubble cloud (registered in ultrasound imaging), but that the lesion formation always consisted of a smaller volume within the bubble cloud. The authors conclude that controlling formation of the bubble cloud is the key to controlling formation of the lesion.

The third study, by Phillips et al., demonstrated the* in vivo* performance of these composite droplets at forming lesions in rat livers [103]. Using and MR-guided focused ultrasound and MR thermometry, the heat delivery and lesion formation in rat livers were assessed after injection of the composite nanoscale droplets. When no agents were administered, no significant lesion formation or heat delivery occurred during a 15 s pulse of 1 MHz ultrasound (4.1 MPa peak negative pressure). In contrast, a single bolus injection of nanoscale droplets enhanced lesion formation over controls out to the last time point tested (95 minutes), and lesions were approximately constant in size at each time point.

Studies are ongoing to compare the* in vivo* performance of these composite droplets to microbubbles in both rat livers and superficial tumors in order to determine the relative benefits in circulation time and control over lesion formation. We are also assessing biodistribution to determine whether the nanoscale droplets are, indeed, able to extravasate at solid tumor sites. The preliminary results showing a sustained ablation-enhancement effect in the liver does not necessarily indicate that droplets have remained in circulation for 95 minutes, as droplet clearance is expected to result in accumulation within the liver. While future studies are needed to determine actual vascular circulation profiles (which depends on both thermal stability and active clearance by the body's immune response), these results do show that the droplets remain thermally stable for at least 95 minutes* in vivo*, despite being composed of two perfluorocarbons with boiling points far below body temperature. This highlights the benefit of designing agents from volatile components in order to optimize performance. If it turns out that the vascular circulation profile is short, this may be improved by altering the encapsulation to produce particles more “stealthy” to the immune response.

One remaining question surrounding PCA use in HIFU ablation is whether it is better to design droplets that vaporize at the same pressures used for HIFU ablation or whether it is better to create droplets that vaporize at higher pressures and use a high-intensity “bubble generation” pulse to seed the bubble cloud before ablation begins (as demonstrated by Zhang and Porter [49]). Our phantom-based studies suggest that so long as the droplet concentration is not too high, control over lesion formation can be maintained even when the vaporization pressures coincide with the ablation pressures. Future comparisons are needed to evaluate tradeoffs between these two concepts of PCA-aided ablation.

### 5.3. Blood-Brain Barrier Permeabilization with Volatile PFC Droplets

Just as reduced vaporization thresholds open new possibilities in diagnostic and molecular imaging, they also open the possibility to use droplets in therapeutic applications that require low ultrasound pressures. Many studies have shown that the blood-brain barrier (BBB) can be transiently permeabilized noninvasively using focused ultrasound in order to enhance drug delivery [9, 10]. The presence of microbubbles enhances this effect and allows delivery of a larger payload, but the ultrasound parameters and microbubble size must be controlled so that no permanent damage is incurred [104]. One of the additional benefits of blood-brain barrier opening with microbubbles is that the acoustic emissions caused by microbubbles cavitating within the ultrasound beam can be passively captured and used as an indicator of BBB opening [105, 106]. Most studies have found that, in the presence of typical bubble distributions and frequencies near 1 MHz, peak negative pressures must remain on the order of 600 kPa or less to avoid hemorrhage and unwanted tissue damage [104].

Similar to the previous studies involving the enhanced focal abilities of droplets versus microbubbles for HIFU ablation, droplet vaporization may be an alternative method of BBB opening provided droplets vaporize at the low pressures required in this therapy. In a study by Chen et al., we collaborated on a proof-of-principle study comparing the performance of DFB droplets to DFB microbubbles (each agent had the same constitutive elements, but one began in the liquid state and the other in the gas state) [107]. Our previous measurements have shown that at frequencies near 1 MHz, DFB droplets vaporize optimally at peak negative pressures near 2 MPa, although a small degree of vaporization is observed at pressures near 1 MPa [91]. Further high-speed imaging experiments revealed that a small amount of vaporization (primarily of large droplets in the distribution) occurs at pressures near 0.45 MPa or higher—which may be sufficient for initial demonstration of BBB opening.

**Figure 12:**
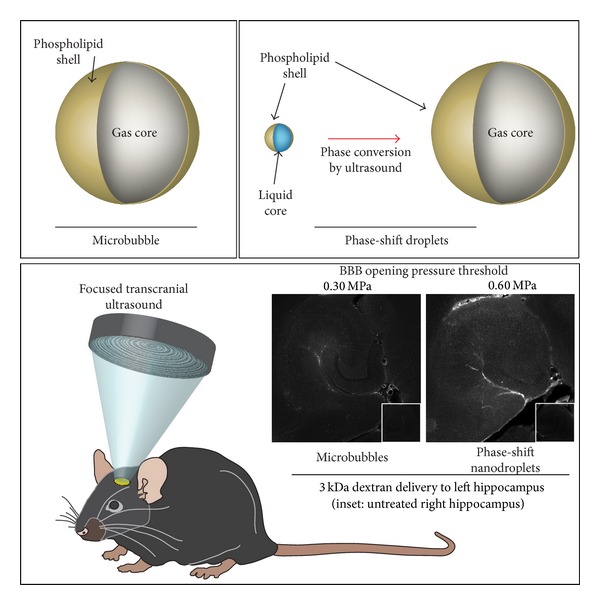
Blood-brain barrier opening with ultrasound contrast agents. When transcranial ultrasound energy is delivered to the left hippocampus of mice, blood-brain barrier permeability can be increased in the presence of both microbubbles and phase-shift droplets. When coinjected with 3 kDa dextran, the fluorescence increase in histologic analysis of the hippocampus reveals successful permeabilization of the BBB as a function of peak negative pressure. Reprinted from [107], Copyright 2013, with permission from Elsevier.

To test this* in vivo*, transcranial focused ultrasound was delivered to the left hippocampus of mice based on previous protocols with microbubbles [108] and the right hippocampus was used as a control. Mice were separated into two groups: one that received a bolus injection of microbubbles and one that received a bolus injection of nanoscale droplets. To compensate for the low vaporization efficiency at these pressures, the administered droplet concentration (5 × 10^9^ particles/mL) was nearly an order of magnitude higher than the bubbles (8 × 10^8^ particles/mL). Though this was a higher dose in number density, the smaller particle size (204 nm diameter for droplets versus 1.36 *μ*m for bubbles) resulted in a significantly lower volume density for the droplet injection (1.054 *μ*L/mL for bubbles versus 0.022 *μ*L/mL for droplets). In both groups, a coinjection of dextran was administered to act as a model drug. Cavitation emissions were captured passively during the therapy in order to correlate with BBB opening results and mice were monitored for 1 hour after sonication. Fluorescence histology of the brain in the treated animals showed that DFB microbubbles were much more effective than DFB droplets at delivering dextran, with BBB opening beginning at peak negative pressures of 0.3 MPa. Droplets were able to successfully open the BBB at pressures of 0.45 and 0.6 MPa, corresponding well with the observed bubble formation* in vitro *at these pressures. The degree of dextran delivery was similar for microbubbles at 0.3 MPa and droplets at 0.6 MPa (Figure 12).

The focal improvement of droplets compared to bubbles is evident in the acoustic emissions data. No significant cavitation is registered for droplets until peak negative pressures reach 0.6 MPa—corresponding with the pressures observed to cause BBB opening. For bubbles, on the other hand, cavitation signal is present at even the lowest pressures used, where no BBB opening is observed. The lack of cavitation signal for droplets is expected to be a result of bubble formation only occurring at the focus—minimizing bubble interaction with the beam elsewhere. Further comparison between the fluorescence and cavitation results corroborates this—providing a higher correlation for droplet-based opening than bubble-based opening. In these studies, no histological damage was observed for BBB opening with droplets, although it was observed for BBB opening with bubbles at 0.6 MPa. The authors conclude that predicting the level of BBB opening based on acoustic emissions may depend on the type of contrast agent being used, but that droplets appear to provide a higher-confidence prediction.

Studies are ongoing to expand on this initial demonstration. Because the DFB agents were chosen for similarity to the conventional agents, they were not optimized to vaporize at the ideal pressures. We are currently investigating use of lower boiling point droplets that vaporize more efficiently and are expected to produce a greater degree of BBB opening. Because droplets are significantly smaller than microbubbles, it also may be possible for droplets to diffuse past the BBB once initial opening is achieved and allow greater drug delivery into the nearby tissue. Here, the vaporization pulse was adopted based on microbubble protocols, but BBB permeabilization with droplets may require droplet-specific pulses to optimize bubble production (such as a short high-intensity pulse followed by a longer pulse of lower intensity). Some aspects of this study require further investigation. For instance, whether the mechanism of BBB opening with droplets is similar to the bubble mechanism or whether the initial expansion phase contributes significantly is currently unknown. If the latter turns out to be the case, then more volatile PFCs such as OFP may be more effective in permeabilizing the barrier. Also, whether the observed effect is primarily a result of the larger droplets in the distribution is currently unknown. Optimization of the droplet size for BBB disruption through size-selection techniques or microfluidics may enhance performance greatly.

### 5.4. *In Vitro* and* In Vivo* Demonstration of Custom PCA Contrast-Imaging Sequences

One of the least-explored aspects of PCAs is their utility for pure contrast generation. The molecular imaging study detailed above (Section 5.1) provides initial evidence that PCAs with reduced thresholds can be used to gain diagnostic information, but much work remains to demonstrate the contrast-providing abilities. For PCA-based diagnostic imaging to succeed in the clinic, it is likely that both activation and imaging must be implemented on a single device (e.g., a single transducer). In order to accomplish this, custom pulse sequences must be developed to maximize the diagnostic information provided. It is important to note that phase-change agents have unique requirements as contrast agents with regard to both imaging and activation, which makes implementation much more challenging than conventional microbubble contrast agents. For example, baseline (preactivation) imaging must be done at pressures low enough so that droplet vaporization does not occur. This requires careful design of both the pulse pressures and the PCAs so that imaging can be performed at pressures that provide reasonable imaging quality. Once it is desired to generate contrast, activation pulses must be delivered at high enough pressures to maximize droplet vaporization, but only in the desired region and in a manner that minimizes bioeffects. This requires careful planning of the pulse spacing and pulse pressure/length. Finally, the imaging (postactivation) must occur quickly enough that the newly generated contrast does not leave the imaging plane and must use pulse pressures low enough to not disturb the resulting bubbles (or cause additional droplet vaporization). For applications such as molecular imaging or for droplets that have extravasated into the interstitial space (as is the focus of much PCA research), bubbles will theoretically remain stable in-plane, and so the postactivation timing is less demanding. However, for nontargeted vascular applications, newly generated bubbles will begin to wash out of the imaging plane with blood flow immediately after being generated. In most applications, this means bubbles must be imaged before leaving the elevational beam width, although this will depend somewhat on the vascular orientation of the target.

To our knowledge, only two demonstrations to date have shown both activation and imaging within a single transducer [99, 109]. The first, our* in vitro* molecular imaging study outlined in Section 5.1, used two independent pulse sequences available on a clinical diagnostic machine and performed imaging and activation at separate time points under static conditions. The second, by Couture et al., consisted of a custom pulse sequence to accomplish a therapeutic task of delivering a fluorescent payload and used the received ultrasound data as confirmation. The latter study demonstrates the potential to develop pulse sequences for droplet-specific tasks using a new generation of highly customizable ultrasound research machines. While theirs investigated a therapeutic goal, the high degree of control over pulse sequencing and data manipulation opens the door to developing custom sequences for PCA-based diagnostic imaging.

**Figure 13:**
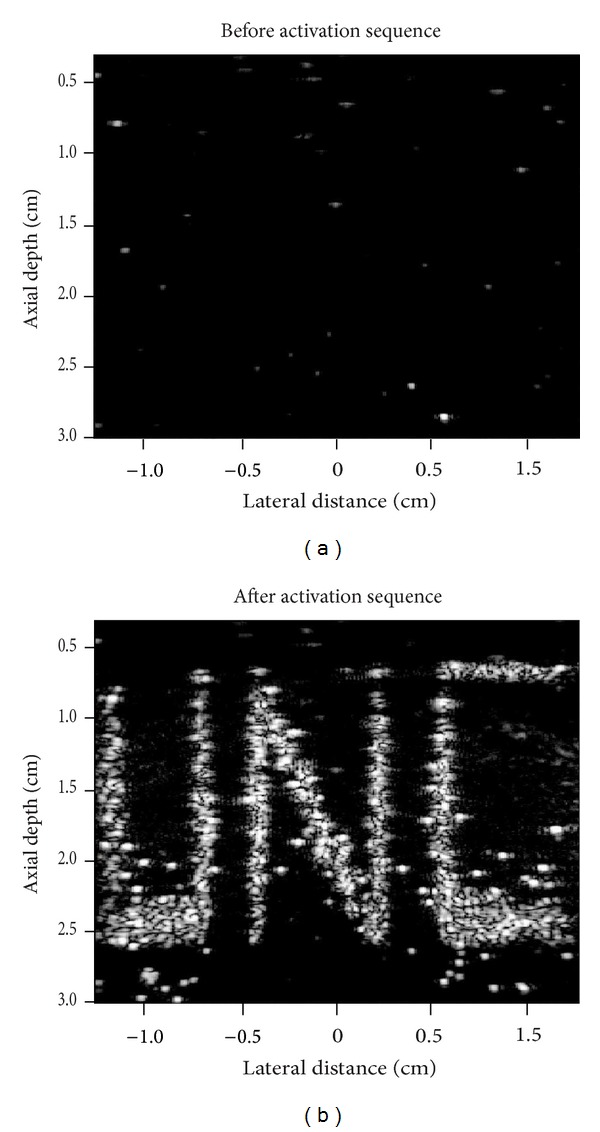
Plane-wave pulse inversion images of a room-temperature water bath containing OFP droplets before (a) the droplet activation sequence and immediately after (b) the final pulse of the activation sequence. The focus of each preprogrammed activation pulse was shifted using electronic steering to create the desired pattern in a left-to-right, bottom-to-top order. Unpublished data courtesy of Jordan Hjelmquist.

**Figure 14:**
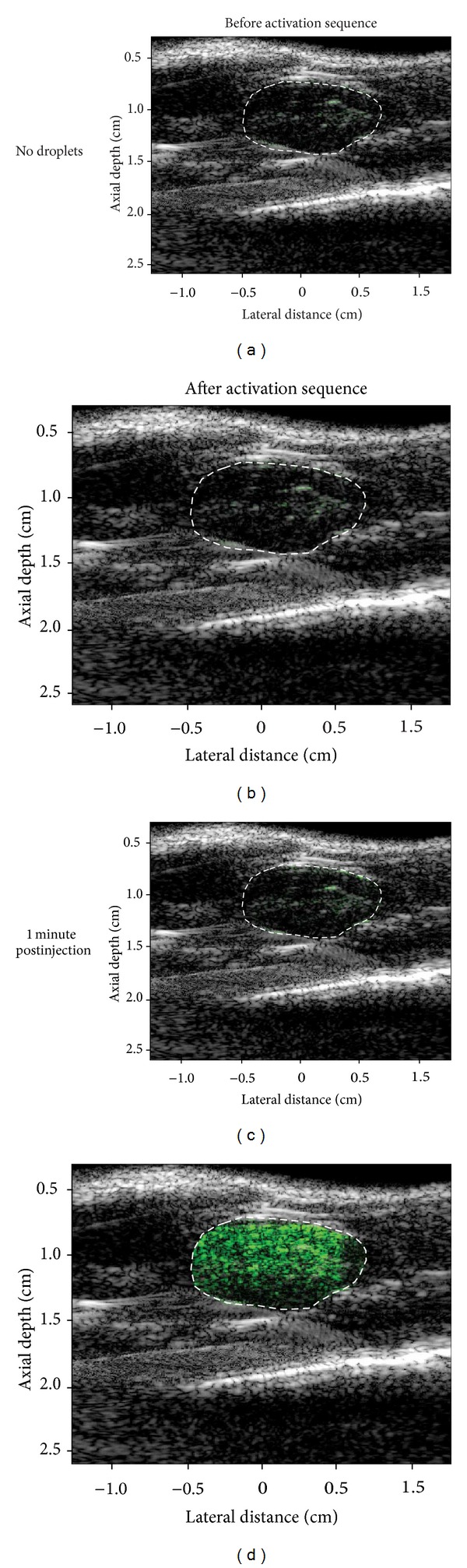
Overlays of contrast-specific intensity (green scale) and traditional B-mode (grey scale) ultrasound scans within the region of interest (white dotted line). When no droplets are present (top row), very little contrast is present before (left) and after (right) the vaporization pulse sequence. When a bolus of OFP droplets is injected, no significant increase in contrast is observed before the activation sequence (bottom row, left), but once the activation sequence is delivered a high level of contrast is visible in the region of the kidney (bottom row, right)—indicating conversion of the droplets to form highly echogenic microbubbles. Unpublished data.

Studies are ongoing in our group to demonstrate this using the Verasonics research ultrasound platform (Verasonics, Inc., Redmond, WA) both* in vitro* and* in vivo*. Using the rat kidney as a vascular target, we have developed PCA-specific sequences that accomplish synchronized activation and imaging in the same plane with high control over timing and pressure (unpublished data). Imaging before and after vaporization is accomplished using a pulse-inversion plane-wave scheme with approximate negative pressures of 0.55 MPa (4.5 MHz, 1 cycle, pressure approximated based on depth-dependent tissue attenuation), and activation is accomplished using electronically steered focused wave pulses (7.5 MHz, 1 cycle) with peak negative pressures near 2.40 MPa (based on approximate tissue attenuation) that are spaced laterally and axially to overlap minimally so that newly generated bubbles are not disturbed highly by the subsequent pulse. The timing between activation and baseline/postactivation imaging (as well as the frame-rate of both imaging states) can be adjusted easily in order to capture contrast after it has been generated. Control over the generation of contrast temporally and spatially with electronically steered focused pulses can be demonstrated* in vitro* by exposing a room-temperature water bath containing approximately 1 × 10^7^ droplets/mL of OFP particles to a preprogrammed pulse pattern (Figure 13). Comparing the baseline to postactivation images reveals that contrast can be generated with high spatial specificity and captured quickly before the bubbles leave the imaging plane. Preliminary* in vivo* results show that OFP droplets (injected as a 120 *μ*L 50% droplet dilution in saline) can be easily activated and imaged with a single transducer and provide a high degree of contrast relative to baseline. After injection, but before activation, no significant contrast increase is observed in the kidney—implying the injected droplets remain in the liquid state while in circulation. When the activation pulse is delivered after 1 minute of circulation, mean image intensity in the imaging plane increases on the order of 20 dB over baseline (Figure 14), and the contrast washes out of the imaging plane over several seconds. Repeating the sequence at different time points allows capture of the circulation profile of the droplets. Preliminary results also suggest that contrast generation with submicron droplets using PFC mixtures much higher in boiling point than DFB result in minimal, if any, contrast generation as the ability to get adequate pressures at depth in tissue to cause vaporization becomes a challenge with the imaging transducer.

Many aspects of this study are currently being refined. For instance, activation must only cover the desired area so that interaction with other tissues is minimized. But to activate well in the desired area, several choices must be made with regard to axial and lateral pulse spacing that also depend on PFC choice (what pressures are actually needed to activate well) and frequency (how much the pressures are attenuated in tissue). The timing that newly generated contrast remains in-plane also depends on factors such as pressure and frequency, and an understanding of how far into the elevational plane droplet vaporization is occurring. Some other factors are currently unexplored. For example, most droplet activation thresholds are developed* in vitro*, but the higher* in vivo* hydrostatic pressures may impact the efficiency of vaporization. The degree of this is not certain, but in combination with the frequency-dependent tissue attenuation, understanding the proper frequencies and pressures needed to induce droplet activation while in circulation will be vital.

Additionally, extensive testing on bioeffects caused by the agents is needed if PCAs are to have a future role in diagnostic imaging. Because of the similarity in composition to clinical microbubble formulations (i.e., phospholipid-encapsulated perfluorocarbons), it is expected that clearance of the particles developed in our studies results in deposition primarily in the liver and spleen, with eventual exhalation of the perfluorocarbon through the lungs [110, 111]. In general, perfluorocarbons have proven to be relatively nontoxic at the small doses used for most medical applications [34, 35, 112]. Most studies on* in vivo* performance of PCAs have noted no adverse effects resulting from administration [31]. However, only a few studies have actually performed dose-dependent survival studies. Most notably, a study by Zhang et al. using microscale droplets of DDFP (mean diameter of 2 *μ*m) observed that changes in blood chemistry and respiratory distress occurred following intravenous injection in a canine model once doses reached 2 × 10^9^ droplets/kg, and doses of 3 × 10^9^ (total perfluorocarbon dose of 0.2 g/kg) caused fatality [67]. The bolus injections used for our preliminary studies investigating PCA use in rodents falls on the same order of magnitude (4 × 10^9^ droplets/kg assuming a 150 g rodent) as these canine upper limits. However, the average droplet diameter (265 nm [91]) is an order of magnitude smaller than those in the study, by Zhang et al., resulting in a much lower total perfluorocarbon dose of 4.7 × 10^−4^ g/kg. Accordingly, none of the preliminary studies to date have resulted in visible symptoms of distress in the animals tested. Future studies will be needed to determine upper dose limits for nanoscale PCAs as well as to evaluate the likelihood of vascular damage cause by droplet vaporization events or from acoustic interactions with the bubbles produced early in the vaporization pulse.

Regardless, these studies show that PCAs can be used effectively as blood-pool contrast agents, which implies the possibility to also develop effective droplet-based molecular imaging sequences and evaluate new vascular applications for PCAs.

### 5.5. Summary

In this section, we show that by combining concepts such as incorporation of volatile compounds with those of tuning droplet size and performance, current therapeutic applications of PCAs can be substantially improved, and new diagnostic and therapeutic applications can be developed.

## 6. Future Directions and Conclusion

In this review, we have highlighted recent progress to improve the performance of PCAs. In our opinion, tailoring droplet characteristics highly to each intended application and abandoning a “one-size-fits-all” approach will be critical in moving phase-change agents toward the clinic. In addition, work with programmable ultrasound research systems to develop ideal activation (and imaging, if the application benefits from it) will be key in demonstrating the unique opportunities phase-change agents provide. It is likely that, due to similarity in composition and applications, the success of PCAs will depend somewhat on the clinical success of microbubbles, which has made slow progress in some countries (including the USA). However, demonstrating success in new applications not possible with microbubbles (such as extravascular imaging/therapy, selective vascular occlusion) and showing the enhanced performance over microbubbles in other applications will help distinguish the two agents and pave an independent path for PCAs. When it becomes time to translate PCAs to the clinic, we are hopeful that the process may be more rapid than required for a completely new agent—since PCAs are in fact just “condensed” microbubbles, and can be produced with an identical formulation to that utilized in currently approved microbubble contrast agents.
